# Human endogenous retrovirus type W envelope expression in blood and brain cells provides new insights into multiple sclerosis disease

**DOI:** 10.1177/1352458512441381

**Published:** 2012-12

**Authors:** Hervé Perron, Raphaëlle Germi, Corinne Bernard, Marta Garcia-Montojo, Cécile Deluen, Laurent Farinelli, Raphaël Faucard, Francisco Veas, Ilias Stefas, Babs O Fabriek, Jack Van-Horssen, Paul Van-der-Valk, Claire Gerdil, Roberta Mancuso, Marina Saresella, Mario Clerici, Sébastien Marcel, Alain Creange, Rosella Cavaretta, Domenico Caputo, Giannina Arru, Patrice Morand, Alois B Lang, Stefano Sotgiu, Klemens Ruprecht, Peter Rieckmann, Pablo Villoslada, Michel Chofflon, Jose Boucraut, Jean Pelletier, Hans-Peter Hartung

**Affiliations:** 1 GeNeuro, Switzerland; 2 GeNeuro-Innovation, France; 3 Doctoral School, University of Lyon, France; 4 Department of Virology, University Hospital, Grenoble, France; 5 Fasteris, Switzerland; 6 APOH-Technologies, France; 7 IRD, Université de Montpellier, France; 8 TNO, Zeist, The Netherlands; 9 Department of Pathology & Dept. of Molecular Cell Biology and Immunology, VU University Medical Centre, The Netherlands; 10 Etablissement Français du Sang, France; 11 Laboratory of Molecular Medicine and Biotechnologies; Don C. Gnocchi ONLUS Foundation,Italy; 12 Department of Biomedical Sciences and Technologies, University of Milan, Italy; 13 Department of Neurology, University Hospital, Grenoble, France; 14 Department of Neurology, Université Paris Est, France; 15 Multiple Sclerosis Unit, Don C. Gnocchi ONLUS Foundation, Italy; 16 Dipartimento Neuroscienze e Scienze Materno-Infantili, Neurology Clinic, University of Sassari, Italy; 17 Clinical Research Unit for MS and Neuroimmunology, Department of Neurology, Würzburg, Germany; 18 Centre for Applied Medical Research, University of Navarra, Spain; 19 Department of Neurology, University Hospital, Geneva, Switzerland; 20 Faculty of Medicine, Marseille, France; 21 Department of Neurology, University Hospital, Marseille, France; 22 Department of Neurology, Heinrich Heine University, Germany

**Keywords:** multiple sclerosis, endogenous retrovirus, HERV-W, MSRV, TLR4, syncytin, biomarker

## Abstract

**Background:**

The envelope protein from multiple sclerosis (MS) associated retroviral element (MSRV), a member of the Human Endogenous Retroviral family ‘W’ (HERV-W), induces dysimmunity and inflammation.

**Objective:**

The objective of this study was to confirm and specify the association between HERV-W/MSRV envelope (Env) expression and MS.

**Methods:**

103 MS, 199 healthy controls (HC) and controls with other neurological diseases (28), chronic infections (30) or autoimmunity (30) were analysed with an immunoassay detecting Env in serum. Env RNA or DNA copy numbers in peripheral blood mononuclear cells (PBMC) were determined by a quantitative polymerase chain reaction (PCR). Env was detected by immunohistology in the brains of patients with MS with three specific monoclonals.

**Results:**

Env antigen was detected in a serum of 73% of patients with MS with similar prevalence in all clinical forms, and not in chronic infection, systemic lupus, most other neurological diseases and healthy donors (*p*<0.01). Cases with chronic inflammatory demyelinating polyneuropathy (5/8) and rare HC (4/103) were positive. RNA expression in PBMC and DNA copy numbers were significantly elevated in patients with MS versus HC (*p*<0.001). In patients with MS, DNA copy numbers were significantly increased in chronic progressive MS (secondary progressive MS vs relapsing–remitting MS (RRMS) *p*<0.001; primary progressive MS vs RRMS –<0.02). Env protein was evidenced in macrophages within MS brain lesions with particular concentrations around vascular elements.

**Conclusion:**

The association between MS disease and the MSRV-type HERV-W element now appears quite strong, as evidenced ex-vivo from serum and PBMC with post-mortem confirmation in brain lesions. Chronic progressive MS, RRMS and clinically isolated syndrome show different ELISA (Enzyme-Linked Immunosorbent Assay) and/or PCR profiles suggestive of an increase with disease evolution, and amplicon sequencing confirms the association with particular HERV-W elements.

## Introduction

When the first descriptions of retrovirus-like particles with reverse-transcriptase (RT) activity in leptomeningeal and macrophage cell cultures from patients with multiple sclerosis (MS) were published^1–3^ they were thought to be related to a new human T-lymphotropic virus (HTLV) supposed to explain analogies between HTLV type 1 associated myelopathy and MS.^4^ After preliminary biological characterization of this MS-associated retroviral element (MRSV),^5,6^ molecular analysis of its genome revealed quite a complex picture^7^ as the MSRV genome had endogenous counterparts in human DNA featuring a Human Endogenous Retrovirus (HERV).^8^ Instead of the expected HTLV virus,^4^ it represented an element from a previously unknown HERV family, now named HERV-W.^9,10^

Endogenous retroviral families present in human DNA apparently entered the germ line of most mammalian species millions of years ago through infectious events with transmissible strains, and have often spread within the species genomes through both Mendelian and non-Mendelian transmission.^11^ Surprisingly, sequencing the human genome revealed that over 40% of human DNA sequences belong to the category of repeated or (retro)transposable elements, including endogenous retroviruses (ERVs), themselves representing 8% of human DNA.^11,12^ The question of their biological significance in physiology and in diseases is therefore of considerable interest and stands at the crossroads of genetics, virology and immunology, as reviewed.^13–15^

HERV particles associated with RT activity were confirmed to be a hallmark of MSRV expression^6,16^ and a reverse transcriptase-polymerase chain reaction (RT-PCR) protocol selective for virion-associated MSRV RNA, was designed to detect its presence in patient’s serum and cerebrospinal fluid (CSF).^17^ Independent studies in MS patients and control populations evidenced MSRV correlations with MS disease and prognosis,^17–21^ including the conversion from relapsing–remitting multiple sclerosis (RRMS) to secondary progressive multiple sclerosis (SPMS) forms,^22^ as with the geographical prevalence of MS in Europe.^23^ Parallel studies evidenced the immunopathogenicity of MSRV virions and further identified MSRV Envelope protein (Env) as being responsible for major pro-inflammatory and superantigenic immunological effects^24–26^ whereas Env antigen was detected in MS plaques in post-mortem brain studies.^19,27,28^ Transactivation of HERV-W promoters by environmental viruses associated with MS was also shown to induce an epigenetic dysregulation of HERV-W pathogenic elements when present in the host’s genome.^15^ Resulting inappropriate activation of the innate immune system by MSRV/HERV-W Env is thereby likely to contribute to the development of certain neuroinflammatory diseases, through the ’Toll-Like Receptor-4 (TLR4) and CD14 co-receptor’ pathway.^25,26^ Elevated expression of Toll-Like Receptor-3 (TLR3) and TLR4 was reported within microgliocytes and astrocytes of MS active lesions^29^ and plasma levels of soluble CD14 were found to be significantly higher in MS patients versus healthy controls (HC).^30^

Toll-like receptors (TLRs) are major pattern recognition receptors (PRRs) that normally have a central role in the initiation of innate immunity against invading microbial pathogens.^31^ TLRs are activated in glial cells and lymphocytes infiltrating the nervous system in response to inflammation caused by infectious agents, tissue injury or autoimmunity.^32^ TLR4 is revealed as particularly critical in neuroinflammation.^33^

The objective of the present study was to evaluate the prevalence of HERV-W expression in MS patients with all disease forms, while evaluating an immunoassay for the Env^34^ as well as reproduction of a quantitative polymerase chain reaction (qPCR) selective for the env gene of HERV-W MSRV subtype.^35^ Therefore, after complementary evaluation of the immunoassay in neurological, infectious or autoimmune diseases and in healthy controls, we have evaluated HERV-W/MSRV env RNA and DNA as ex-vivo biomarkers in patients with MS. For this purpose, MS patients from multiple European centres and controls without medical history potentially affecting HERV-W expression patterns were recruited for this study focused on MS. It consequently required HC for studying an HERV-W pathological expression that made no assumption of an exclusive association with MS. Indeed, this association is already known not to be exclusive for MS.^34,36^

## Patients and methods

For the preliminary evaluation of the HERV-W immunoassay, serum samples were obtained from HC and patients with MS, and other neurological or non-neurological diseases. For the European study with immunoassay and PCR reproduction a blinded panel of MS patients from different geographical areas was recruited. To study the ex-vivo expression in peripheral blood, we collected sera and mononuclear cells (PBMC) samples from MS patients in Hospital Neurological Departments and from healthy blood donors in Transfusion Centres. For the preliminary evaluation of an ELISA(Enzyme-Linked Immunosorbent Assay)^34^ on various patients and controls a total of 167 sera were analysed blindly. Samples comprised (i) other neurological diseases (OND): 20 patients with epilepsy, chronic polymyositis, primary cerebral tumour, sciatica, Guillain–Barré Syndrome, stroke, multisystem atrophy, facial palsy, amyotrophic lateral sclerosis (ALS), cerebral metastasis (lung cancer), Leigh’s disease, traumatic medullar lesion, cerebral abcess (listeria), together with eight patients with chronic inflammatory demyelinating polyneuropathy (CIDP); (ii) non-neurological diseases (NND): 15 patients with chronic hepatitis B virus infection, 15 with chronic hepatitis C virus infection, 30 with systemic lupus erythematosus (SLE); and (iii) healthy blood donors: 60 HC. In the following multicentre study, ELISA immunoassay was performed on blinded samples in an external laboratory (APOH technologies, Montpellier, France). Non-blinded reference sera (10 negative HCand 10 positive MS) were sent for technical validation and to determine the normal threshold (cut-off value) of the assay. Blinded samples from 26 additional HC and from 74 randomly selected patients with definite MS according to the McDonald criteria were then tested (Table 1A).^37^ Samples from 14 patients with clinically isolated syndrome (CIS), according to the same McDonald criteria, were also tested (Table 1B). No significant difference (Chi-Square test, *p*>0.05) in age and gender was found between MS and HC, but CIS cases could not be matched for these criteria as intrinsically representing a rather homogeneous younger population. Additional non-blinded series of control sera from 103 healthy blood donors were tested thereafter. PBMC were tested according to a previously described qPCR and RT-PCR specific for the MSRV subtype of the HERV-W family^35^ and further replicated with an optimized protocol. When samples did not allow RNA and/or DNA extraction in sufficient quantity or quality, they were not tested and the corresponding patients were not analysed in the test series. The study designs, protocols, documentation and the sample coding preserving anonymity were all approved by the ethical committees of the participating institutions. All patients gave written informed consent before inclusion. Samples from healthy blood donors were obtained following the specific procedures of blood banks preserving anonymity. Technical protocols, methods and materials are further detailed in the Supplementary Material.

**Table 1.** Clinical and treatment data from the multicentre European study.

		***n***	Median	SD	Range
**(A) Patients with multiple sclerosis (n=74)**					
**Age (years)**			47.53	13.46	20–77
**Disease duration**			14.31	9.68	1–43
**EDSS**			4.75	2.1	1–8
**Gender (male/female)**		29/45			
			Dis. dur.*/EDSS	Dis. dur.*/EDSS	Dis. dur.*/EDSS
**Disease form**	Relapsing–remitting	26	6.52 / 2.38	5.34 / 0.68	0–22 / 1–3.5
	Secondary progressive	39	19.32 / 6.13	8.77 / 1.14	7–43 / 3.5–8
	Primary progressive	9	12 / 5.5	7.91 / 2.01	1–26 / 1.5–7
**Non treated**		19	–	–	–
**Treated with:**	Interferon-beta	24	–	–	–
	Cyclophosphamide	10	–	–	–
	Methotrexate	1	–	–	–
	Mitoxantrone	9	–	–	–
	Azathioprine	14	–	–	–
	Glatiramer acetate	5	–	–	–
Treated with combinations**		17	–	–	–
**(B) Patients with clinically isolated syndrome (*n*=14)**					
**Age**			29.21	9.31	18–47
**EDSS**			1.13	0.77	0–3.5
**Gender (male/ female)**		3/11			
**Non treated**		8	–	–	–
**Treated with**	Interferon-beta	1	–	–	–
Treated with combinations**		1	–	–	–

* Dis. dur.: disease duration (years).

** Association of at least two of the above mentioned treatments. In the case of multiple sclerosis relapse, blood samples were drawn prior to the beginning of the therapy.

EDSS: Expanded Disability Status Scale

## Results

### HERV-W-Env antigen detection in serum

In the pilot study with the colorimetric read-out, results from the HC population (*n*=60) were used to determine the cut-off value of the assay (see the Supplementary Material). Accordingly, 23 of the 29 MS patients had positive MSRV Env antigenemia, and six were negative. Among eight CIDP patients five were positive. The remaining OND patients (*n*=20) were negative. None of the 30 samples from cases of chronic hepatitis B virus infection or chronic hepatitis C virus infection was found to be positive. Polyreactive sera from 30 patients with SLE, positive for anti-DNA, anti-nucleus antibody and rheumatoid factor, did not yield positive results either. Results from all MS patients were significantly different from all OND patients (Mann–Whitney rank sum test: *p*<0.001). Results from the complete MS group were not significantly different from the isolated CIDP subgroup (Mann–Whitney rank sum test: *p* = 0.053).

**Figure 1.**
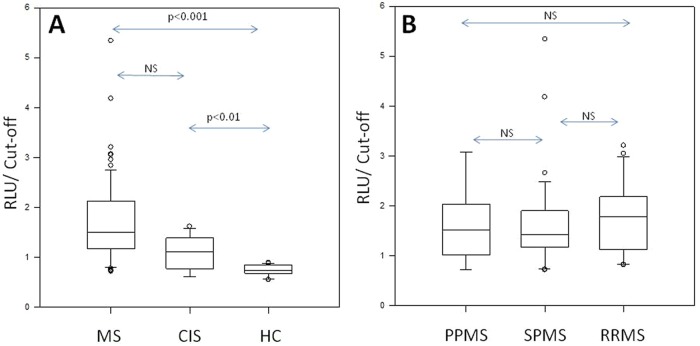
Human Endogenous Retroviral family ‘W’ (HERV-W) envelope (Env) detection. (A) HERV-W Env antigenemia in multiple sclerosis (MS) and clinically isolated syndrome (CIS) serum compared to normal population; (B) HERV-W Env antigenemia in serum of different MS clinical forms. Arrows with ‘*p*’ values represent the statistical significance of the observed difference between patients versus blood donors (Mann–Whitney rank sum test); NS=Not Significant (*p*>0.05). *Y*-axis: the value is expressed as the ratio of the measured ELISA (Enzyme-Linked Immunosorbent Assay) value to the cut-off value from the normal population of healthy blood donors (mean+2SD of healthy controls, HC); values >1 are positive. PPMS: primary progressive MS; RLU: relative light unit; RRMS: relapsing–remitting MS; SPMS: secondary Progressive MS.

As presented in Table 2 for the following series from multiple European centres, 59 out of 74 (79.7%) non-selected MS cases had positive values for HERV-W Env protein, with luminometric read-out providing a greater range than in the pilot series, but none of the 36 HC (26 blinded and 10 non-blinded as reference controls) had positive values. The difference in values and prevalence between MS patients and HC was highly significant (Figure 1A; Mann–Whitney rank sum test: *p*<0.001). Most CIS patients (9/14; 64.3%) had positive antigen detection and, despite lower values than MS patients, were significantly higher than HC (Figure 1A; Mann–Whitney rank sum test: *p*<0.01). In a larger series of blood donors made for addressing the question of possible carriers in the healthy population, few positive cases were detected with the same luminometric read-out (4/103; 3.8%, data not shown). These results were significantly different from MS and CIS patients (Mann–Whitney rank sum test: *p*<0.001), but indicated the existence of apparently healthy or asymptomatic carriers. Analysis of Env immunoassay values from different forms of MS (primary progressive MS (PPMS), SPMS and RRMS) showed no significant difference between these subgroups (Figure 1B). In the majority of tested patients receiving specific therapy such as interferon, cyclophosphamide, mitoxantrone and/or corticosteroids, the presence of circulating HERV-W Env protein did not seem to be significantly affected; a similar absence of influence on results was seen for age and gender.

**Table 2.** HERV-W Env protein detection in multiple sclerosis (MS) and clinically isolated syndrome (CIS) serum compared to normal population.

	Positive	Negative	Highest value	Mean value	Mean of positives	Tested (*N*)	Statistics*
**MS**	80%	20%	5.34	1.70	1.92	74	***p* < 0.001**
**CIS**	64%	36%	1.62	1.11	1.30	14	***p* = 0.002**
**Blood donors**	0%	100%	0.90	0.75	NA	26	

* Mann–Whitney rank sum test: statistical significance of the observed difference between patients versus blood donors.

The maximum statistical value of normal population (cut-off) has been determined from the series of reference serum of healthy blood donors (cut-off = mean + 2SD), the value is expressed as the ratio of the measured ELISA (Enzyme-Linked Immunosorbent Assay) value to the cut-off value from normal population of blood donors.

### HERV-W Env DNA and RNA quantification in PBMC by real-time qPCR

A recent PCR technique selective for the HERV-W subtype most homologous to MRSV,^35^ targeting the gene encoding the retroviral Env, was adapted to a Light-Cycler PCR platform (Roche) for the analysis of RNA and DNA from PBMC samples, in an independent laboratory (Department of Virology, CHU Grenoble). These samples corresponded to the multicentre serum series. CIS samples as well as several MS cases had no available, or too few, PBMC, which precluded their inclusion in the PCR series (see Table 3). The present study was also replicated by another team on separate aliquots of frozen PBMC from the same samples sent to GeNeuro-Innovation PCR facilities, on another qPCR platform (Biorad; see Supplementary Material).

**Table 3.** Human Endogenous Retroviral family ‘W’ (HERV-W) envelope (Env) (multiple sclerosis associated retroviral element (MSRV) type) expression in MS peripheral blood mononuclear cells compared to normal population.

	Positive	Negative	Highest value	Mean value	Mean of positives	Tested (*N*)	Statistics*
**(A) Increase of MSRV-envelope RNA expression in MS**							
**MS**	40%	60%	109.32	2.83	11.20	58	***p* < 0.001**
**Blood donors**	4%	96%	1.74	0.23	NA	26	
**(B) Increase in MSRV-envelope DNA copy number in MS**							
**MS**	40%	60%	34.77	3.61	7.94	62	***p* < 0.001**
**Blood donors**	4%	96%	1.37	0.46	NA	26	

* Mann-Whitney Rank Sum Test

The maximum statistical value of normal population (cut-off) has been determined from the series of reference PBMC of healthy blood donors (cut-off= mean + 2SD), the value is expressed as the ratio of the measured polymerase chain reaction (PCR) RNA or DNA copy number to the cut-off value from normal population of blood donors (mean+2SD).

#### PBMC RNA

**Figure 2.**
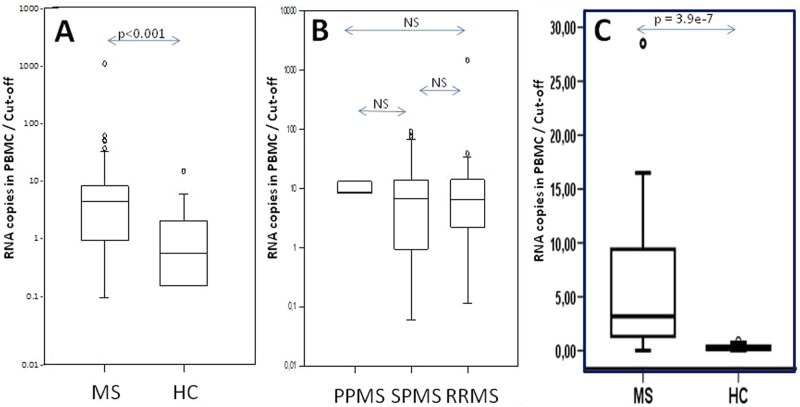
Human Endogenous Retroviral family ‘W’ (HERV-W) envelope (Env) (multiple sclerosis associated retroviral element (MSRV) type) RNA expression. (A) MSRV Env RNA expression in MS peripheral blood mononuclear cells (PBMC) compared to normal population; (B) MSRV Env RNA expression in MS PBMC of different MS clinical forms; (C) MSRV Env RNA expression in MS PBMC compared to normal population (results from internal replication study in bold). Arrows with ‘*p*’ values represent the statistical significance of the observed difference between patients versus healthy blood donors (Mann–Whitney rank sum test); NS=Not Significant (*p*>0.05). *Y-*axis: the results are presented as the ratio between the measured copy number and the threshold value determined from the mean value plus twice the standard deviation of copy numbers from all healthy controls (HC) (mean+2SD of HC). Results with higher copy number than the threshold of the normal individuals have values above ‘1’. PPMS: primary progressive MS; RRMS: relapsing–remitting MS; SPMS: secondary progressive MS.

A real-time quantitative polymerase chain reaction (RT-qPCR) evidenced a significant difference in the positivity and in the expression levels of PBMC MSRV RNA between MS patients and HC (Mann–Whitney rank sum test: *p*<0.001; Table 3A and Figure 2A). The highest MS RNA levels were two Logs above the threshold of HC. MS and HC mean values differed by one Log. Among HC, one individual’s value was above the threshold (outlier).

As for antigenemia results, when RNA copy numbers from different MS forms were compared, no significant difference in MSRV Env RNA expression could be evidenced between PPMS, SPMS and RRMS (Figure 2B). Moreover, no correlation with treatment, including interferon, could be evidenced.

The replication study performed under different conditions yielded similar results, which confirmed a significant difference between MS patients and HC (Figure 2C). Of note, the outlier from HC seen in Figure 2A could not be included for the replication study presented in Figure 2C, as a sufficient quantity of PBMC was not available for the purpose of RNA extraction. Sufficient DNA quantity being available, it was included in the DNA replication study presented below.

#### PBMC DNA

**Figure 3.**
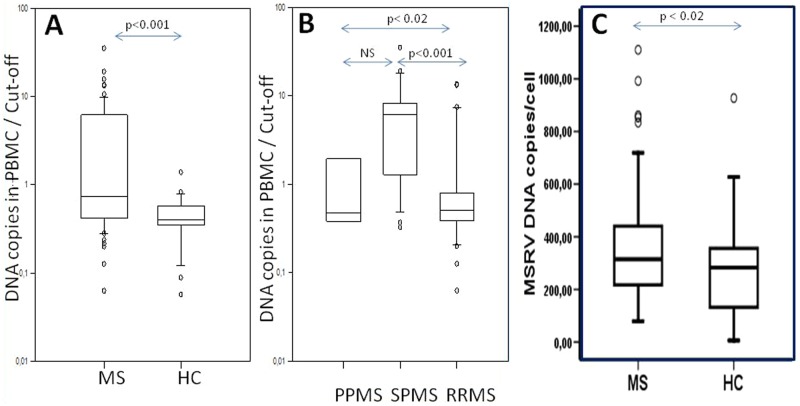
Human Endogenous Retroviral family ‘W’ (HERV-W) envelope (Env) (multiple sclerosis associated retroviral element (MSRV) type) DNA copy number variation. (A) MSRV Env DNA copy number in MS peripheral blood mononuclear cells (PBMC) compared to normal population; (B) MSRV Env DNA copy number in MS PBMC of different MS clinical forms; (C) MSRV Env DNA copy number in MS PBMC compared to normal population (results from internal replication study in bold). Arrows with ‘*p*’ values represent the statistical significance of the observed difference between patients versus blood donors (Mann–Whitney rank sum test); NS=Not Significant (*p*>0.05).*Y* axis: the results are presented as the ratio between the measured copy number and the threshold value determined from the mean value plus twice the standard deviation of copy numbers from all healthy controls (HC) (mean+2SD of HC). Results with higher copy number than the threshold of the normal individuals have values above ‘1’. PPMS: primary progressive MS; RRMS: relapsing–remitting MS; SPMS: secondary progressive MS.

The same specific primers and the probe in qPCR adapted for DNA amplification confirmed the originally reported findings,^35^ consisting of an increase of MSRV DNA copy number within PBMC for MS patients versus HC. This highly significant difference (Mann–Whitney rank sum test: *p*<0.001;Table 4B) is illustrated in Figure 3A. Of note, the same HC outlier in the RNA series had a DNA copy number slightly above the threshold. However, contrary to results from Env and RNA detection, a significant difference was evidenced between MS forms, with chronic progressive MS (SPMS or PPMS) having higher DNA copy number than RRMS (Mann–Whitney rank sum test: *p*<0.001 for SPMS and *p*<0.02 for PPMS; Figure 3B). As most SPMS patients had longer disease duration and higher Expanded Disability Status Scale (EDSS) than in the other forms (Table 1), a correlation with DNA results was sought for; a trend existed but did not reach statistical significance. Elsewhere, an eventual influence of treatment could not be evaluated due to small numbers with each type of therapy, with the exception of interferon treatment for which a too small number of untreated RRMS patients precluded comparison between untreated or treated RRMS and SPMS. In addition, the type of interferon, its dosage, an eventual association with other treatments and the duration of treatment differed between RRMS patients. A potential influence of treatment on these different DNA copy numbers between RRMS and SPMS could therefore not be evaluated. The replication study performed under different conditions, e.g. with a standard monocopy ’RNase P’ gene providing results as an average DNA copy number/cell, yielded similar results and confirmed a significant increase in MS cases versus HC (Figure 3C).

**Table 4.** Human Endogenous Retroviral family ‘W’ (HERV-W) envelope clones obtained from peripheral blood mononuclear cells by standard polymerase chain reaction (PCR) with primers used in the quantitative PCR.

Clone reference	Status	DNA PCR	DNA PCR	RNA RT-PCR	RNA RT-PCR
		Number of clones	Sequences aligned with probe	Number of clones	Sequences aligned with probe
**(A) Multiple Sclerosis (MS) patients**					
1	PPMS	6	1	4	3
2	PPMS	5	2	9	4
3	SPMS	6	1	6	5
4	SPMS	6	4	9	1
5	RRMS	13	5	8	5
6	RRMS	7	2	5	5
	**Total:**	**43**	**15**	**41**	**23**
**(B) Healthy controls**					
A	Healthy	10	0	9	2
B	Healthy	10	4	7	2
C	Healthy	5	1	2	0
D	Healthy	5	2	0	0
E	Healthy	5	1	8	0
F	Healthy	4	0	2	0
	**Total:**	**39**	**8**	**28**	**4**

PPMS: primary progressive MS; RRMS: Relapsing–remitting MS; RT-PCR: reverse transcriptase-polymerase chain reaction; SPMS: secondary progressive MS.

### Sequence analyses of amplicons from MS patients versus HC

Products were cloned and sequenced in order to address the specificity of the quantification obtained with the present PCR technique (see Methods, Supplementary Material). The PCR products from six representative MS cases (two RRMS, two SPMS and two PPMS) with RNA and/or DNA copy number above the threshold and six HC below the threshold (except one individual with slightly elevated copy numbers) were selected for the purpose (Table 4). In order to avoid any mistake in reassembling irrelevant fragments with overlapping sequences by deep sequencing of short stretches in a complex mixture of variants, PCR products were cloned. This provided a representative panel of inserts with DNA and RNA amplicons from each PBMC sample. Vectors with inserts were sequenced on both strands to eliminate any ambiguous base.

PBMC DNA and RNA respectively yielded 43 and 41 sequences in MS samples, as well as 39 and 28 in HC (Table 4). The sequence of the probe used for qPCR was then aligned with these sequences. Sequences showing significant alignment (Figure S3, Supplementary Material) corresponded to the amplicons determining the copy numbers measured by this probe in the qPCR reaction: their percentage reflected the differences observed with qPCR (Table 4 and Figure S3, Supplementary Material): 56% (23/41) in MS RNA versus 14% (4/28) in HC (chi-square test: *p*<0.001).

**Figure 4.**
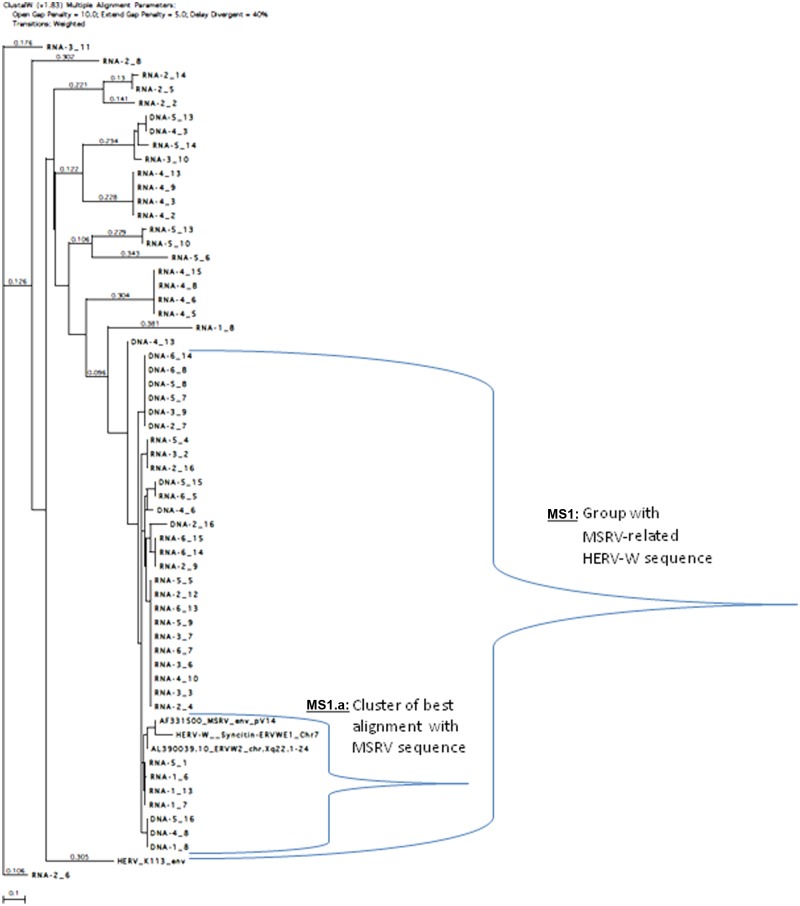
Alignment tree of multiple sclerosis (MS) RNA and DNA clones with reference multiple sclerosis associated retroviral element (MSRV), Human Endogenous Retroviral family ‘W’ (HERV-W) (ERVWE1 and ERVWE2) sequences and distant HERV-K envelope (Env) sequence. ClustalW (v1.83) multiple alignment parameters: open gap penalty=10.0; extend gap penalty=5.0; delay divergent=40%. Transitions: weighted. The calculated distance between branched clones is indicated by the horizontal length separating them (reference length unit represented at the bottom), or by the numbers on the lines (when not proportional). The total distance between two clones is the addition of these measures.

Detailed analysis was performed by aligning these clones with HERV-W reference clones (MSRV Env, ERVWE1 encoding Syncytin on chromosome 7) and a distant Env gene from another HERV family (HERV-K113-Env), using clustalW (v1.83) multiple alignment (Figure 4). Significant homology with MSRV Env reference sequence (AF331500) was evidenced for 39 MS RNA sequences and for 15 MS DNA sequences. Twenty-three of the 39 RNA sequences were significantly aligned with the probe sequence (Figure S3, Supplementary Material) whereas all 15 DNA sequences were aligned, which suggests the existence of relevant RNA variants in MS samples not detected by the probe. This implies a probable underestimation of MSRV RNA copy number in MS samples with the present conditions and possible avenues for future technical optimizations.

Clones obtained from MS RNA and DNA with high homology to MSRV sequences were identified (‘MS1’group in Figure 4) but a cluster of clones (‘MS1.a’) was revealed closest to the MSRV sequence and to highly homologous HERV-W copies (ERVWE2 or ERVWE1). The ’MS1.a’ cluster may represent the most accurate cluster of MSRV sequences from MS.

**Figure 5.**
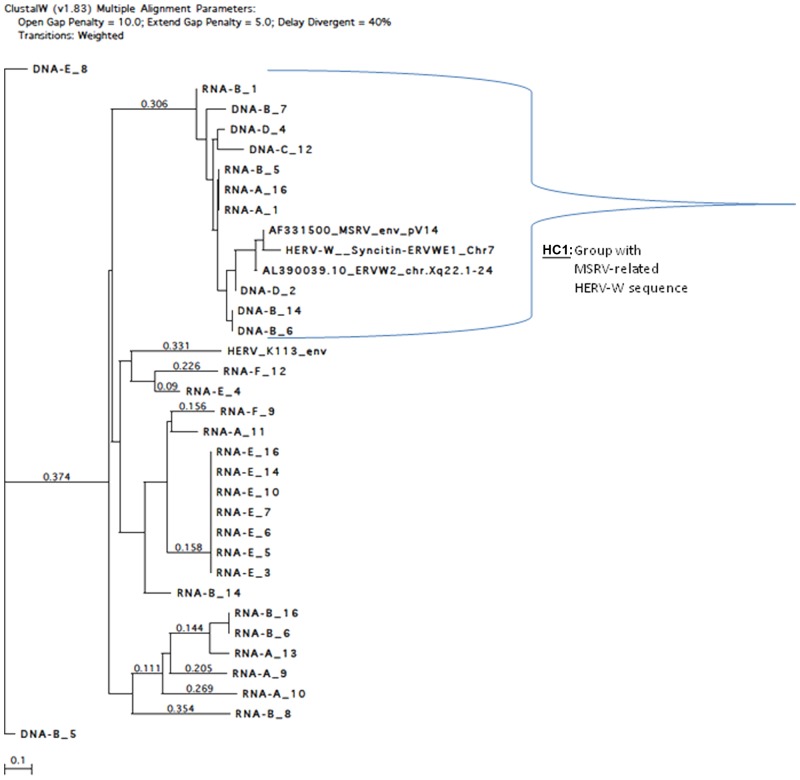
Alignment tree of healthy controls (HC) RNA and DNA clones with reference multiple sclerosis associated retroviral element (MSRV), Human Endogenous Retroviral family ‘W’ (HERV-W) (ERVWE1 and ERVWE2) sequences and distant HERV-K envelope (Env) sequence. ClustalW (v1.83) multiple alignment parameters: open gap penalty=10.0; extend gap penalty=5.0; delay divergent=40%. Transitions: weighted. The calculated distance between branched clones is indicated by the horizontal length separating them (reference length unit represented at the bottom), or by the numbers on the lines (when not proportional). The total distance between two clones is the addition of these measures.

An MSRV-like group is found in HC RNA and DNA (‘HC1’ group in Figure 5), but the distances between clones are greater than in MS cases and the number of homologous sequences is smaller. Quite importantly, further analyses evidenced differences between MS and control sequences (see Figure S4, Supplementary Material). Thus, the great majority of MS clones that significantly aligned altogether matched with the detection probe previously used to quantify ’MSRV subtype’, but only a minority did so in HC. This provides a qualitative rationale for the quantitative difference previously measured between MS and HC cases in the ’probe-targeted’ qPCR (Figures 2 and 3).

### MS brain immunohistology

**Figure 6.**
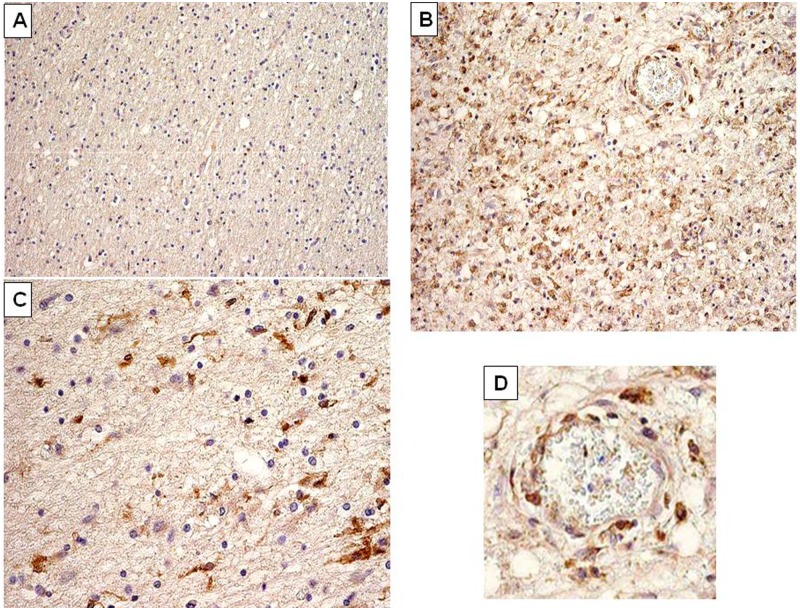
Multiple sclerosis (MS) brain immunohistology evidencing Human Endogenous Retroviral family ‘W’ (HERV-W)/MS associated retroviral element (MSRV) envelope (Env) protein in MS brain lesions. (A) Normal appearing white matter (magnification ×10); (B) early active lesion with numerous Env-positive cells stained in brown (magnification ×40); (C) edge of active lesion, showing less numerous Env-positive cells stained in brown (magnification ×40); (D) detail of (B), magnifying a vascular element within the lesion with perivascular Env-positive cells stained in brown. These cells present morphological features and perivascular dissemination of macrophages. Immunohistological staining is representative of anti-HERV-W Env specific staining in different sections. Brown cells represent positive cells, significantly labelled by anti-Env monoclonals (examples with GN-mAb_03 are presented in this figure).

**Figure 7.**
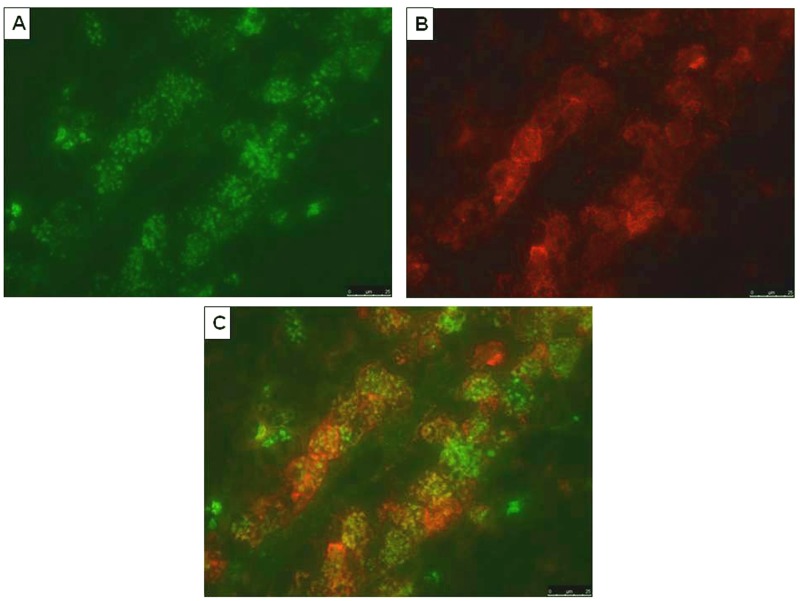
Confirmation of envelope (Env) expression in perivascular macrophages by double Immunostaining. Sections were selected from a lesion area including a vascular element with evidence of perivascular macrophage cuffs infiltrating the parenchyma, as in Figure 6D. (A) Perivascular macrophages labelled with anti-Env GN-mAb_05 mouse monoclonal (1:100) and revealed in green with fluorescent labelled secondary antibodies; (B) immunoactive macrophage labelled with anti-MHC class II LN3 antibody (1:50) and revealed in red with fluorescent labelled secondary antibodies (same slide); (C) merge of (A) and (B) immunolabelling evidencing the co-localization of both anti-Env and anti-MHC II antigens in macrophage cells from a perivascular cuff, in the same sample from an active multiple sclerosis lesion. In cells where the two antigens are co-expressed, orange, yellow or light-brown colour is seen when the two antigens have superimposed location with varying proportion, thus modulating the merged colours. Few cells also seem to express a single antigen, with single colour.

To determine the expression of HERV-W Env we selected a small collection of eight well-characterized MS brain samples containing chronic active lesions (*n*=5) and early active lesions (*n*=3) (Figure S1, Supplementary Material). Classification of lesion staging was based on standard histopathological staining for inflammatory cells (anti-MHC class II) and myelin (anti-PLP).^38^ HERV-W Env immunoreactivity was virtually absent in normal appearing white matter (NAWM) as seen in Figure 6A. In active lesions containing abundant macrophages and activated microglia we observed HERV-W Env expression in infiltrated macrophages throughout the brain parenchyma and in perivascular infiltrates (Figures 6B and 6D). Moreover, activated microglial cells at the rim of chronic active lesions are HERV-W Env-immunopositive (Figure 6C). Importantly, macrophages and activated microglia were consistently stained with all three anti HERV-W Env monoclonals, both in active lesions as well as in the rim of chronic active lesions. Double staining with HLA-DR confirmed that HERV-W Env localized to macrophages in a perivascular infiltrate (Figures 7A–7C). Only a few cells seem to have single antigen expression (Env or HLA-DR), which confirms that this expression is not involving all macrophages and is not occurring in macrophages exclusively but in the greatest majority within perivascular cells of active plaques. Altogether, 8/8 MS brains tested revealed positivity for HERV-W Env in demyelinated lesions with similar staining patterns detected by the same specific monoclonal antibodies. No such staining was seen in corresponding NAWM and non-neurological controls (*n*=3).

## Discussion

The present study has focused on peripheral blood (serum and PBMC) as a suitable source of samples for ex-vivo studies and repeated sampling for, e.g., clinical trials and patient monitoring. Therefore, CSF was not considered appropriate. The choice of the target protein was also justified by its immunopathogenicity^24–26^ and by its ability to induce neuroinflammatory models with anti-Myelin autoimmunity as in experimental allergic encephalomyelitis (EAE) in mice, as now evidenced and further validated in pre-clinical regulatory studies (Hervé Perron, oral communication, European Antibody Congress, Geneva, Switzerland, 2011).

The preliminary study focusing on HERV-W Env detection in serum was set up for an evaluation of specificity, after RNA PCR studies had repeatedly confirmed an association with MS.^39^ Positive Env detection was observed in a large majority of MS patients. Env was not detected in OND but their limited number cannot preclude further detection in certain OND groups or in other chronic diseases, as can be seen here in a few cases with CIDP. Consistently, a recent study evidenced significant HERV-W Env antigenemia in a group of Schizophrenic patients with inflammatory conditions.^34^ Nonetheless, no positivity was found in chronic infections (chronic hepatitis B or C) or in polyreactive sera from another autoimmune disease, SLE. After a previous study on patients with rheumatoid arthritis showing no significant association with HERV-W/MSRV detection,^40^ these data confirm the non-ubiquitous nature of this endogenous retroviral expression in infectious or inflammatory diseases, be it a circulating protein, extracellular virion or PBMC RNA. Indeed, as illustrated here with the CIDP cases, HERV-W Env protein and RNA/DNA from this MSRV-subtype are not specific biomarkers for MS, neither are they ubiquitous nor simply reflecting infectious, inflammatory or autoimmune diseases.

The purpose of the next steps of the present study was to focus on their detailed expression in MS, whereas another study focusing on CIDP is now ongoing and should be followed by other complementary prospective evaluations. Thus, this multicentre study aimed at specifying HERV-W/MSRV Env, RNA and DNA detection profiles in non-selected MS patients. It also provided a double replication study of the previous qPCR results^35^ and of immunoassay detection of Env in a larger number of MS sera (after the preliminary study including groups with various diseases). Therefore, this part of the study focused on MS disease versus healthy status. Our first PCR series were performed with the protocol defined by Mameli et al.^35^ on the Light-Cycler platform. Our internal replication series reproduced once more the HERV-W/MSRV RNA and DNA results with significant increase in MS. Elsewhere, the observed DNA copy number in chronic progressive MS significantly differing from RRMS now requires further study, in particular with larger numbers of cases with PPMS. Concerning Env protein detection by immunoassay, technical limitations due to both 2A12A5 antibody and APO-H capture have led to the present development of a sandwich antibody format with specific anti-Env monoclonals on a newly developed platform. Therefore, the major information provided by the present study consists of the reproducible data on HERV-W Env detection in MS.

A highly significant increase in RNA and DNA MSRV-like copy numbers replicates previous observations^35^ but the presence of circulating HERV-W Env protein in serum from most MS cases and its detection with monoclonal antibodies targeting three different domains within MS brain lesions is reported here for the first time. Thus, previous^19,27,28^ and present observations by immunohistology of dominant expression in brain macrophage cells within MS perivascular lesions are consistent with the observed findings in MS PBMC. Macrophages originate from blood monocytes that are part of PBMC and migrate within and from brain lesions through the bloodstream^41^ which suggests that they could be a major source of MSRV expression in PBMC. As now well described, brain macrophages originate from blood or lymph through a low but constant recirculation that is sharply increased in inflammatory conditions.^42^ These data also fit with studies on MS blood macrophages, in which retroviral particles with RT activity were repeatedly detected.^1,3,6^

Interestingly, our evaluation of the same qPCR technique on whole blood samples showed no such difference with either RNA or DNA. This may be due to the important dilution of the ‘specific and elevated signal’ probably limited to few circulating macrophages among numerous non-productive cells with HERV background within whole blood.

HERV-W/MSRV expression is also known to occur in EBV-positive B-lymphocytes^6^ but their presence in PBMC is limited to very few B-cells, as evidenced by the low EBV genome copy levels in the same PBMC samples (Figure S2B, Supplementary Material). Therefore, in blood samples, an increase in HERV-W/MSRV DNA copy number in MS PBMC may rather indicate a genomic retroviral amplification attributable to MSRV-associated RT activity in MS macrophages.^43^ Indeed, unlike RNA expression and protein detection, an increase in DNA copy number per cell is not expected from a normal gene expression but fits with such retroviral enzyme activity. From a virological point of view, this requires further investigation on the more-or-less replicative activity of a peculiar MSRV/HERV-W element and on its eventual complementation or interference with other more-or-less defective HERV copies.

From a biomarker point of view, an increase in specific HERV-W/MSRV Env DNA copy may correlate with other disease parameters than RNA or protein expression, as suggested by the significant difference between chronic progressive MS versus RRMS for DNA copies (Figure 3B), which is not observed for RNA and antigen detection (Figures 1B–2B). As explained in the Results section, an eventual influence of interferon treatment in the present RRMS series on MSRV DNA copy number, compared to non-interferon treated SPMS, could not be evaluated. Nonetheless, the absence of a difference for protein or RNA expression between RRMS and SPMS does not support the hypothesis that interferon in RRMS could explain a lower DNA copy number than in SPMS in our present cohorts. However, our recruitment dominantly selected hospitalized patients who were admitted on MS clinical criteria, which should exclude most patients with long-term remission or with stabilization and response to treatment. One study showed an effect of interferon on HERV-W RNA expression during a one year follow-up with treatment-responding patients and an elevation of RNA copies after a drop in treatment efficacy.^44^ Indeed, a different population of patients was recruited, which consisted of RRMS patients from routine visits for treatment follow-up. In our case, inclusion of patients was linked to MS disease, but not to treatment of RRMS only. In parallel, the group of CIS patients with first neurological episode, included in the antigen detection series only, yielded about 60% of positives. This was not significantly different from definite MS but values revealed a much lower mean titer and narrower range than in MS (Figure 1A). In most cases, CIS presents with a first neurological episode announcing the clinical onset of a future evolution towards MS. Thus, the present data support previous prognostic results of HERV-W/MSRV detection in CIS or equivalent isolated episodes.^45^ Altogether, these observations indicate that bioclinical correlations may exist, which should be specified by further studies including longitudinal follow-up and should be useful for therapeutic monitoring.

MS (or CIS) patients with negative or normal results for HERV-W/MSRV biomarkers may correspond to a subgroup(s) of patients with other pathogenic pathways not involving HERV-W elements, or to patients in remission or without disease activity at sampling.^44^ Beyond the probable existence of different etiopathogenic mechanisms causing MS, it suggests that heterogeneity in MS studies may also arise from patients presenting different etiopathogenic origins of MS symptoms, as consistently observed in the 10-year survey of MS patients divergent for HERV-W expression at clinical onset.^22^

Sequence analyses provided a rationale for the quantitative difference measured by the present qPCR technique. It also evidenced a cluster of clones from MS, most homologous to reference MSRV Env sequence, as well as co-amplification of known defective HERV-W copies (Figure 4). This is expected in DNA, as such a multicopy gene family does not allow perfect selectivity of copies sharing sequence homology with one another. These can also be co-activated in disease together with MSRV expression, through common MSRV transactivators or activation pathways.^46–48^ They may also be (over-) expressed in MS but not transactivated in HC, and can interfere with coding RNAs as reported^49,50^, and obviously explain background RNA copy detection in most HC (Figure 5). A consistent interpretation of the present results with other apparently diverging ones^35,51^ is that several HERV-W copies can be co-activated by the mere co-factor having transactivated HERV-W copies and/or by the MSRV ongoing expression itself. However, the defective gag and pol genes within, e.g., the HERV-W copy encoding Syncytin (HERV7q Env gene or ERVWE1 locus) excludes its role in RT activity or in virion particle production. Moreover, the antibody used to claim Syncytin detection (6A2B2) in MS brains may recognize Env from MSRV subtype, but not from Syncytin in non-denaturing conditions.^52^

**Figure 8.**
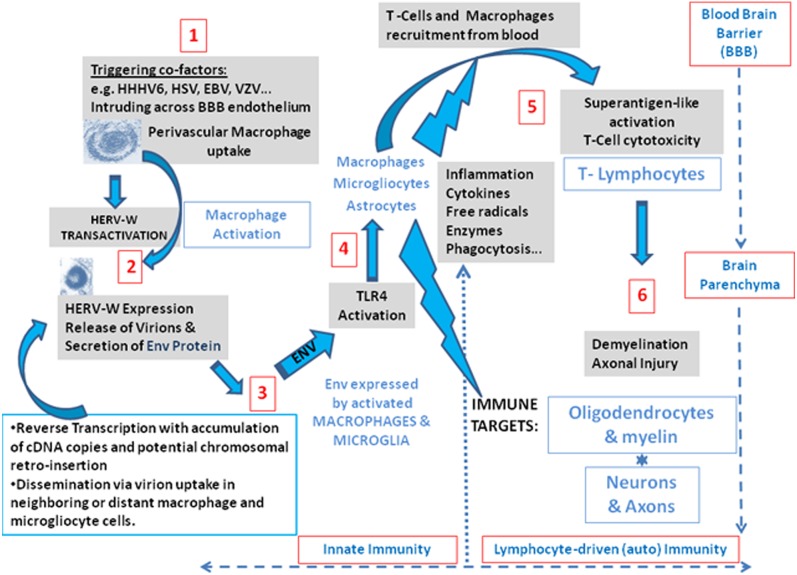
Global scenario of an immunopathogenic and neuroinflammatory pathway involving Human Endogenous Retroviral family ‘W’ (HERV-W) envelope (Env) expression as a pivotal element between infectious virus co-factors and the neuroimmune pathogenic cascade.

As is now better characterized with three antibodies having distant and independent epitopes used for Env detection in MS brain, the presence of a full-length Env is supported. Env-antigen expression is again confirmed to occur in MS lesions, particularly within infiltrated perivascular macrophages, which is also consistent with previous observations of cultured macrophages, isolated from MS blood.^3,6^ These results thereby support the parallel between early results showing retroviral expression in MS^1,3,17,53^ and this pathogenic HERV-W expression of MSRV subtype sequences and Env. It therefore confirms the accuracy of the rationale for its ex-vivo detection in peripheral blood, as in the present study. This protein has major immunopathogenic properties as evidenced by original studies,^24,26^ the features of which are also seen ex-vivo in immune cells from MS patients.^25,54^ This thereby means that such an immunopathogenic protein can be pivotal to MS immunopathogenesis when present in lesion sites, secreted by active macrophage cells as described,^3^ and re-circulating in MS blood with significantly increased DNA copy number. Such additional DNA is commonly produced by RT from retroviral genomes, whether it is endogenous or exogenous, and RT activity was the first evidenced biomarker of this HERV expression in MS cells.^1,3^ These results together with the many others accumulated over the past two decades indicate that this MSRV subtype of the HERV-W family is likely to play a role in initiating and fueling a pathogenic ’chain reaction’ causing MS.^55^ A scenario depicting HERV-W activation by environmental co-factors in perivascular white-matter macrophages and the subsequent activation of an immunopathogenic cascade in CNS parenchyma is presented in Figure 8. HERV-W/MSRV expression with Env pathogenicity there appears to be pivotal between (i) environmental and incidental infectious co-factors and (ii) the downstream neuroimmune cascade causing MS lesions.

Based on the present observations and on the preclinical efficacy of an anti-Env neutralizing monoclonal antibody in MS-like EAE animal models induced by HERV-W Env, as evoked above in this discussion, a humanized antibody is now to be evaluated in clinical trials with MS patients (Clinical Phase I has been achieved). More generally this domain of HERV association with complex human neurological diseases is now emerging with recent evidence of an association of HERV-K family elements and ALS.^56^
